# Effect of Improving Dietary Quality on Arterial Stiffness in Subjects with Type 1 and Type 2 Diabetes: A 12 Months Randomised Controlled Trial

**DOI:** 10.3390/nu8060382

**Published:** 2016-06-21

**Authors:** Kristina S. Petersen, Peter M. Clifton, Natalie Lister, Jennifer B. Keogh

**Affiliations:** School of Pharmacy and Medical Sciences & Sansom Institute for Health Research, University of South Australia, GPO Box 2471, Adelaide SA 5000, Australia; kristina.petersen@mymail.unisa.edu.au (K.S.P.); peter.clifton@unisa.edu.au (P.M.C.); natalie.blanch@mymail.unisa.edu.au (N.L.)

**Keywords:** diabetes, arterial stiffness, pulse wave velocity, augmentation index, central blood pressure, diet, nutrition

## Abstract

People with diabetes have accelerated arterial stiffening. The aim of this study was to determine the effect of increasing fruit, vegetable and dairy intake for 12 months on carotid femoral pulse wave velocity (cfPWV), augmentation index (AIx), and central blood pressure (cBP), compared to a usual diet control, in people with type 1 and type 2 diabetes. In a 12 months randomised controlled trial, cfPWV, AIx and cBP were measured every 3 months. The intervention group received dietary counselling to increase consumption of fruit (+1 serving/day; 150 g/day), vegetables (+2 servings/day; 150 g/day) and dairy (+1 serving/day; 200–250 g/day) at baseline, 1, 3, 6 and 9 months. The control group continued on their usual diet. One hundred and nine participants were randomised and 92 (intervention *n* = 45; control *n* = 47) completed. At 3 months, fruit (184 g/day; *p* = 0.001) and dairy (83 g/day; *p* = 0.037) intake increased in the intervention group compared with the control group but this increase was not maintained at 12 months. After adjustment for baseline measurements there was no time by treatment effect for central systolic or diastolic BP, AIx or cfPWV. A time effect existed for AIx which modestly increased over time. Peripheral diastolic BP and central pulse pressure were improved in the intervention group compared with the control group at 12 months. In the cohort with type 1 and type 2 diabetes, improving dietary quality by increasing consumption of fruit, vegetables and dairy did not improve cBP, AIx or cfPWV, compared with a control group continuing on their usual diet, after 12 months.

## 1. Introduction

Individuals with type 1 and type 2 diabetes are two to three times more likely to develop cardiovascular disease (CVD) compared to the general population [1,2]. In Australia, in 2010 approximately 30% of all deaths in people with type 1 and type 2 diabetes were due to CVD [3].

Improving dietary quality may be a strategy to reduce the burden of CVD in people with diabetes. Epidemiological studies show that better dietary quality is associated with lower rates of CVD in the general population [4] and in people with type 1 and type 2 diabetes [5,6]. We have previously shown that people with type 1 and type 2 diabetes have similar dietary quality, as defined by a priori indices, to age, sex and body mass index (BMI) matched non-diabetic subjects [7]. Furthermore, when dietary quality was improved for 12 months it was shown that common carotid artery intima media thickness (CCA IMT) regressed to a greater extent than was observed in the control group [8].

Carotid femoral pulse wave velocity (cfPWV) is considered the gold standard for determining arterial stiffness [9] and is an independent predictor of cardiovascular and all-cause mortality [10]. Furthermore, the addition of cfPWV to conventional Framingham risk factors improves 10 years CVD risk prediction in those at intermediate risk of CVD [10]. Augmentation index (AIx) is another indirect measure of arterial stiffness and has been associated with cardiovascular events and total mortality [11]. People with type 1 and type 2 diabetes have accelerated arterial stiffening when compared to their non-diabetic counterparts [12,13].

We have shown that greater consumption of reduced fat dairy and vegetables was correlated with lower cfPWV in a population with type 1 and type 2 diabetes [14]. Previous epidemiological studies in non-diabetic populations suggest that fruit, vegetables and dairy intake may improve PWV and/or AIx [15,16] but randomised controlled trials have failed to support this observational evidence. The aim of this study was to determine the effect of increasing fruit (+1 serving/day; 150 g/day), vegetable (+2 servings/day; 150 g/day) and dairy (+1 serving/day; 200–250 g/day) intake on cfPWV, AIx and central blood pressure (cBP) over a 12 months period, compared to a control group continuing on their usual diet, in people with type 1 and type 2 diabetes. These data are secondary endpoints from a trial that has previously been reported [8].

## 2. Material and Methods

### 2.1. Study Design

A 12 months randomised controlled trial was conducted as previously described [8]. This analysis comprises a sub-group (*n* = 109) whereby cBP, AIx and cfPWV were measured. Ethics approval was obtained from the University of South Australian Human Research Ethics Committee and participants provided written informed consent. The trial was registered with the Australian New Zealand Clinical Trials Registry (ACTRN12613000251729). Subjects were above 18 years of age with diagnosed type 1 or type 2 diabetes for any duration managed with diet, oral hypoglycaemic agents (OHA) and/or insulin. Recruitment was conducted from August 2012 until December 2013 from a database of volunteers, public advertisements and a recruitment company (Intuito Market Research, Adelaide, SA, Australia). Exclusion criteria were: unstable CVD requiring active intervention, heart failure, significant renal impairment (eGFR < 30 mL/min), liver disease, cancer or allergic/intolerant/dislike of fruit, vegetables or dairy.

In a parallel design, participants were randomised to either the intervention group (improved diet quality) or the control group (continue usual intake) using an online generated balanced random number allocation sequence [17] stratified by diabetes type and sex, by a person independent of the study. The details of the dietary intervention have previously been reported [8]. Briefly, nutritional counselling to increase consumption of fruit (+1 serving/day; 150 g/day), vegetables (+2 servings/day; 150 g/day) and dairy (+1 serving/day; 200–250 g/day), regardless of usual intake, was provided to the intervention group. The control group were not given any dietary advice.

At 1, 3, 6, 9 and 12 months, participants met with a qualified dietician and completed a checklist to determine on how many days of the week they complied with the intervention. A random spot urine sample was provided by the participants at the 3 monthly appointments for measurement of potassium and creatinine. A food frequency questionnaire (FFQ) was administered at 3 and 12 months to determine if food and nutrient intake had changed from baseline.

### 2.2. Measurements

At 3 monthly intervals cfPWV, AIx and cBP were measured. Anthropometric measures, peripheral blood pressure (BP), blood lipids, fasting glucose, C reactive protein (CRP) and spot urinary sodium and potassium excretion were also measured every 3 months. All of the measurements were completed after an overnight fast and the operator completing the vascular measurements was blinded to the participant’s randomisation.

### 2.3. Central Blood Pressure and Augmentation Index

A SphygmoCor^®^ XCEL (AtCor Medical, West Ryde, Sydney, Australia) was used to measure cBP and AIx. A cuff was placed over the brachial artery on the right arm and measurements were completed after the participants had been quietly resting for 5 min. Three consecutive measurements were taken and the average calculated. All of the measurements were taken by one operator with a CV of 4.2% (*n* = 28).

### 2.4. Pulse Wave Velocity

A SphygmoCor^®^ XCEL (AtCor Medical, West Ryde, Sydney, Australia) was used to measure cfPWV. The tonometer was placed on the right carotid artery and the cuff on the right femoral artery. A 10 s recording of the carotid-femoral waveform was taken. Three measurements were performed at each time-point and an average was taken. The measurements were taken by two operators; the intra-observer CVs were 4.2% (*n* = 28) and 7.3% (*n* = 11), respectively and the inter-observer CV was 5.0% (*n* = 18).

### 2.5. Anthropometric Measurements

Height was measured using a stadiometer (SECA, Hamburg, Germany) to the nearest 0.1 cm while barefoot/flat footwear. Weight was measured to the nearest 0.05 kg using calibrated electronic scales (SECA, Hamburg, Germany) while the participants were barefoot/wore light footwear and wore light clothing.

### 2.6. Peripheral Blood Pressure

Peripheral BP was measured using an automated sphygmomanometer (SureSigns VS3; Philips, North Ryde, Sydney, Australia) once the participant had been seated for 5 min. A normal sleeve (16 × 52 cm) was used for an arm circumference of 24–32 cm and a large sleeve (16 × 70 cm) for an arm circumference of 32–42 cm. A minimum of four consecutive readings were taken at 1 min intervals. The first reading was discarded and the following three consistent measurements, i.e., systolic BP within a range of 10 mmHg, were used.

### 2.7. Biological Measurements

#### 2.7.1. Spot Urine Sample

A spot urine sample was provided by participants when they attended the University of South Australia. Analysis of sodium, potassium, creatinine and albumin was done by SA Pathology (Adelaide, SA, Australia). The albumin to creatinine ratio was calculated from one spot urine sample at baseline to determine the presence of micro-albuminuria.

#### 2.7.2. Fasting Blood Sample

A fasting venous blood sample was taken at each time-point by a trained phlebotomist. Total cholesterol, HDL cholesterol, triglycerides, CRP and glucose were measured using a Konelab 20XTi automatic analyzer (Thermo Electron Corporation, Louisville, CO, USA) with reagents from ThermoFisher Scientific (Melbourne, Australia). LDL cholesterol was calculated using the Friedewald formula ((total cholesterol − HDL cholesterol) − (triglycerides × 0.45)) [18]. Serum MMP-7 was measured in duplicate by ELISA (Quantikine Human Total MMP-7, R & D Systems, Minneapolis, MN, USA) according to the manufacturer’s instructions. The inter-assay CV was 5.6%.

#### 2.7.3. HbA1c

The participants were asked to provide the pathology report from their most recent haemoglobin A1c (HbA1c) measurement or the result was sourced from their general practitioner or the pathology company.

#### 2.7.4. Dietary Intake

Dietary intake was measured using the electronic version of the Dietary Questionnaire for Epidemiological Studies version 2 FFQ, as previously described [19].

### 2.8. Statistical Analysis

Data are presented as mean ± standard deviation (SD) or median (interquartile range) depending on the distribution. Data were checked for normality using Shapiro-Wilk and Kolmogorov-Smirnov values. Independent samples *t* tests were used to determine between group differences at baseline for continuous variables and chi squared tests were used for categorical variables. Mixed effect modelling was used to determine between group changes over time and *post hoc* analyses were adjusted for multiple comparisons using the Bonferroni method. For the vascular measurements the results of the mixed effect models are presented as both unadjusted and adjusted for baseline measurements. The twelve months change in anti-hypertensive medication, lipid lowering medication, OHA and insulin were calculated using the following formula: mean (percentage dose change from baseline for each medication)/number of different medications [20]. Independent samples *t* tests were used to determine if there was a difference in medication change between the groups at 12 months. In addition, participants were categorised into the following groups for anti-hypertensive medication, lipid lowering medication, OHA and insulin: medication increased, decreased or no change and chi squared tests were conducted to determine if there was any difference between the groups. A per protocol analysis was conducted to determine if there was any difference in vascular measurements between participants that achieved any increased in fruit, vegetable and dairy consumption at 3 or 12 months and those that decreased consumption. This study was powered on CCA IMT which was the primary outcome but a *post-hoc* power analysis showed that with 78 participants we had 80% power (*p* < 0.05) to detect a 1.1 m/s difference in cfPWV between the groups. With 92 participants we had 80% power (*p* < 0.05) to detect a 9/6 mmHg difference in central systolic and diastolic blood pressure. All of the analyses were performed using SPSS (version 19, 2010, SPSS Inc., Chicago, IL, USA). Statistical significance was set at *p* < 0.05.

## 3. Results

### 3.1. Subjects

One hundred and nine participants were randomised (intervention *n* = 55; control *n* = 54). The characteristics of the participants are presented in Table 1. There were no statistically significant differences in age, anthropometric measures, sex, diabetes type and time since diagnosis, BP, lipids, CRP, glycaemic control or medication prescription between the groups at baseline. Ninety two participants (intervention *n* = 45; control *n* = 47) completed the 12 months study.

### 3.2. Dietary Intake and Compliance

Food and nutrient data measured using the FFQ are available in the online supplemental material (Table 1 and Table 2). Compliance with the intervention measured using the FFQ showed that there was a significant time by treatment effect for fruit (*p* = 0.001) and total dairy consumption (*p* = 0.001) and a non-significant time by treatment effect for vegetable intake (*p* = 0.056). *Post-hoc* testing showed that fruit intake was 184 g/day (95% CI 113, 254 g/day) greater in the intervention group compared with the control group at 3 months (*p* = 0.001); there was no significant difference at 12 months. The intervention group consumed 83 g/day (95% CI 5, 60 g/day) more dairy at 3 months compared with the control group (*p* = 0.037) and this was accounted for by higher yoghurt intake (49 g/day; 95% CI 19, 80 g/day; *p* = 0.002). At 12 months the control group reported a higher consumption of dairy products (90 g/day; 95% CI 14, 166 g/day; *p* = 0.02) due to greater milk consumption (97 g/day; 95% CI 28, 166 g/day; *p* = 0.006). A significant time by treatment effect existed for fibre, calcium, potassium and magnesium. A time effect was present for vegetable and legume (*p* = 0.038), fresh fruit (*p* = 0.03) and yoghurt consumption (*p* = 0.017). At 12 months vegetable and legume intake was 24 g/day higher than at baseline in the whole group (95% CI −1, 49 g/day; *p* = 0.065) and consumption of fresh fruit had increased by 47 g/day (95% CI −3, 67 g/day; *p* = 0.07).

A time effect was observed for the sodium to potassium ratio which increased, see online supplemental material (Table 3). No time by treatment effects existed.

Participants reported that the extra serving of fruit and dairy were consumed on a median of 5.5 days per week throughout the study and at 9 months, participants reported that the extra serve of fruit was consumed on 7 days per week. The two extra servings of vegetables were consumed 5.5 days per week until 9 months and 7 days per week at 12 months.

### 3.3. Blood Pressure and Vascular Measurements

AIx, cfPWV, cBP and peripheral BP measures are presented in Table 2. Measurements of cfPWV were completed on 78 participants (intervention *n* = 36; control *n* = 42) due to technical difficulties because of obesity. A borderline non-significant time effect was observed for peripheral systolic BP (*p* = 0.053) and diastolic BP was reduced over time (*p* = 0.027) with a non-significant time by treatment effect (*p* = 0.053). *Post-hoc* testing showed that diastolic BP was reduced by 1.8 mmHg (95% CI −3.6, −0.06 mmHg; *p* = 0.037) at 6 months compared with baseline; pairwise testing showed no other significant differences between any of the time-points. Central systolic BP and diastolic BP were reduced over time (*p* = 0.001), however there was no time by treatment effect. In the whole group the reduction in central systolic and diastolic BP at 12 months was 5.3 mmHg (95% CI −1.4, −9.2 mmHg; *p* = 0.002) and 3.0 mmHg (95% CI −0.5, −5.4 mmHg; *p* = 0.009), respectively. In addition, central pulse pressure was lowered over time (*p* = 0.003) with no time by treatment effect (*p* = 0.71). After adjustment for the baseline value there were no significant time effects for peripheral or central BP but a time by treatment effect existed for peripheral diastolic BP and central pulse pressure. *Post-hoc* testing showed that at 12 months peripheral diastolic BP was 3.6 mmHg (95% CI −6.4, −0.7) lower in the intervention group compared with the control group (*p* = 0.015). There was no significant difference in central pulse pressure between the groups at any of the time points.

AIx (*p* = 0.002) and central augmented pressure (*p* = 0.02) however increased with time and there was no time by treatment effect. *Post-hoc* testing showed that compared with baseline AIx was increased at 3 (2.7%; 95% CI −0.01, 5.5%; *p* = 0.051), 6 (5.1%; 95% CI 1.5, 8.7%; *p* = 0.001), 9 (2.9%; 95% CI 0.2, 5.5%; *p* = 0.03) and 12 months (2.9%, 95% CI 0.2, 5.5%; *p* = 0.02) compared with baseline in the whole group. Similarly, there was a time effect (*p* = 0.048) for cfPWV with a small increase observed but no time by treatment effect. *Post-hoc* testing showed no statistically significant difference in cfPWV between any of the time-points. After adjustment for baseline values a time effect was present for central augmented pressure and AIx but the time effect present for cfPWV was attenuated to non-significance after adjustment for baseline cfPWV. *Post-hoc* testing showed no significant difference in central augmented pressure or augmentation index between the groups at any of the time-points.

At three months 15 participants increased fruit, vegetables and dairy consumption and 66 participants decreased consumption. At 12 months 20 participants increased consumption of fruit, vegetables and dairy and the remaining 69 decreased consumption. Per protocol analyses showed no between group differences for any of the outcome measurements.

### 3.4. Weight and Biochemistry

Weight, total cholesterol, LDL and HDL cholesterol, triglycerides and glucose remained unchanged over time and there was no time by treatment effect, see Table 3. A time effect existed for CRP (*p* = 0.016) which decreased to a small degree, see Table 3. MMP-7 was measured at baseline and 12 months in a subgroup of the cohort (*n* = 54) and there was no change over time or by treatment, see Table 3.

### 3.5. Change in Medication

There was no statistically significant difference between the groups for the change in medication dose or the number of participants that increased, decreased or did not change their anti-hypertensive medication (82% of participants’ dose was unchanged), lipid lowering medication (89% of participants dose was unchanged), OHA (78% of participants dose was unchanged) and insulin (83% of participants dose was unchanged).

## 4. Discussion

In this cohort of individuals with type 1 and type 2 diabetes cBP and arterial stiffness measures were not improved in the intervention group, compared with the control group at 12 months. Fruit (184 g/day) and dairy intake (83 g/day) were increased at 3 months in the intervention group, but this increase was not maintained at 12 months. There was a border-line non-significant increase in vegetable intake in the intervention group (*p* = 0.056). Central systolic and diastolic BP and pulse pressure were improved over time in the whole cohort, although after adjustment for baseline values the time effect was attenuated to non-significance for cBP. After adjustment for baseline measurements there was a significant reduction in peripheral diastolic BP in the intervention group compared with the control group and a time by treatment effect existed for central pulse pressure. There was no time by treatment effect for AIx or cfPWV but there was a time effect for AIx such that it modestly increased (or deteriorated) over time.

In this study there was a time by treatment effect present for central pulse pressure such that there was a non-significant reduction in the intervention group at 12 months. It has been shown that a 10 mmHg increase in central pulse pressure increases the risk of a cardiovascular event by ~14% [11]. After adjustment for baseline values there was no significant change in cBP over the 12 months study but AIx increased (or worsened) in the absence of any change in cfPWV. This suggests that there was no change in arterial stiffness and the change in AIx was driven by a change in the wave reflection.

Fruit and vegetables have previously been shown to have BP lowering properties which may be explained by their nutritional composition [21]. However, there are a lack of studies investigating the effects of fruit and vegetables or their compounds on cBP. Jennings et al. [22] showed in a cross-sectional study that greater anthocyanin intake is associated with lower central systolic blood pressure. In this study central pulse pressure was improved in the intervention group compared with the control group in the absence of a change in central systolic or diastolic blood pressure. The findings of this study suggest that increasing consumption of fruit and vegetable by small amounts (approximately one third of a serve) which is achievable for free-living people may improve central pulse pressure.

The unchanged cfPWV and MMP-7 levels suggest that vascular remodelling did not occur. MMP-7 acts on collagen and elastin to cause degradation of the extracellular matrix resulting in structural changes and increased arterial stiffness [23]. A recent prospective study with a 4.2 years follow-up, conducted in a cohort with type 2 diabetes, showed that HbA1c during follow-up predicted cfPWV. A greater reduction in HbA1c during the first year of follow-up was associated with less of an increase in cfPWV during follow-up [24]. The authors of this study speculated this finding may be explained by lower advanced glycation end product (AGE) formation with better glycaemic control. AGEs have been shown to be positively associated with cfPWV in subjects with type 1 diabetes [25]. The results of the trial by Ferreira et al. [24] suggest that we may not have observed an effect on cfPWV because glycaemic control was not altered.

A previously conducted randomised controlled trial of people at high risk of CVD with habitually low intake of fruit and vegetables, also showed that greater intake of fruit and vegetables (+2, +4 and +6 portions/day above habitual intake of high or low flavonoid fruit and vegetables) did not affect cfPWV or AIx after 18 weeks, once the data was adjusted for heart rate [26]. A recent randomised controlled trial showed that supplementation with 350 mg of magnesium for 6 months reduced cfPWV by 1 m/s. In the current study the extra serves of fruit, vegetables and dairy provided approximately 80–90 mg of magnesium [27]. Intervention trials examining the effect of dairy on arterial stiffness are lacking.

Dietary intake reported in the FFQ showed that intake of fruit and dairy increased in the intervention group, compared with the control group at 3 months; however, this was not maintained at 12 months. In the compliance checklists the participants reported consuming the extra serves of fruit, vegetables and dairy on greater than 5 days per week throughout the 12 months intervention period. However, the change in fruit, vegetable and dairy intake derived from the FFQ data did not correspond with these checklists, especially for dairy. There was no difference in the urinary sodium to potassium ratio or potassium to creatinine ratio by treatment. Surprisingly the sodium to potassium ratio increased over the 12 months in both groups. This suggests the increase in sodium from dairy products outweighed the increase in potassium from fruit and vegetables despite the FFQ computed intakes.

This study is limited by the poor compliance of the participants with the intervention as shown by the FFQ although biomarkers of fruit, vegetables and dairy intake were not measured to provide an objective measure of compliance. In addition, this work comprises analyses of secondary endpoints and *Post-hoc* power analyses show we had reduced power to detect effects. In this study dietary intake changed in the whole cohort over the study period which may have reduced our capability to detect between group changes. Finally, random spot urine samples taken on one occasion were used to estimate sodium and potassium intake.

## 5. Conclusions

In a cohort with type 1 and type 2 diabetes improving dietary quality by increasing consumption of fruit, vegetables and dairy did not improve cBP, AIx or cfPWV, compared with a control group continuing on their usual diet, after 12 months. Peripheral diastolic BP and central pulse pressure were improved in the intervention group compared with the control group at 12 months.

## Figures and Tables

**Table 1:** Baseline characteristics of the participants that were randomised.

Characteristic	Intervention Group (*n* = 55)	Control Group (*n* = 54)	*p* Value ^1^
Age (years)	57 ± 12	58 ± 12	0.64
Weight (kg)	101 ± 19	99 ± 24	0.61
Height (m)	1.7 ± 0.1	1.7 ± 0.1	0.79
BMI (kg/m^2^)	34.5 ± 6.7	33.4 ± 7.1	0.41
Sex *n* (%)			0.91
Male	32 (58)	32 (59)	
Female	23 (42)	22 (41)	
Diabetes type *n* (%)			0.75
Type 1	5 (9)	4 (7)	
Type 2	50 (91)	50 (93)	
Diagnosed with diabetes (years)	9 ± 8	10 ± 9	0.59
Type 1	21 ± 8	30 ± 10	
Type 2	8 ± 7	8 ± 7	
Smoking status *n* (%)			0.34
Never smoked	22 (40)	29 (54)	
Past smoker	30 (55)	3 (5)	
Current smoker	3 (5)	22 (41)	
Smoking pack years (years) ^2^	10 ± 15	10 ± 16	0.96
Prescribed anti-hypertensive medication *n* (%)	35 (64)	34 (63)	0.94
Prescribed lipid lowering medication *n* (%)	31 (56)	33 (61)	0.62
Diabetes treatment *n* (%)			0.57
None	13 (24)	9 (17)	
OHA	28 (51)	27 (50)	
Insulin	6 (11)	5 (9)	
OHA + Insulin	8 (14)	13 (24)	
Presence of microalbuminuria ^3^ *n* (%)	11 (20)	6 (11)	0.22
Peripheral systolic blood pressure (mmHg)	128 ± 13	130 ± 15	0.54
Peripheral diastolic blood pressure (mmHg)	74 ± 11	71 ± 9	0.12
Total cholesterol (mmol/L)	4.1 ± 1.2	3.7 ± 1.1	0.16
HDL cholesterol (mmol/L)	1.2 ± 0.4	1.3 ± 0.3	0.46
LDL cholesterol (mmol/L)	2.3 ± 1.1	2.0 ± 0.8	0.08
Triglycerides (mmol/L)	1.3 ± 0.8	1.2 ± 1.2	0.70
Glucose (mmol/L)	8.0 ± 3.3	7.7 ± 2.9	0.60
hsCRP (mg/L)	2.7 ± 2.5	3.1 ± 2.8	0.50
HbA1c			
(%)	7.3 ± 1.4	7.4 ± 1.6	0.68
(mmol/mol)	56 ± 16	57 ± 17	0.68

All values are mean ± SD or *n* (%) where specified; ^1^ Intervention vs. control independent samples *t* test; ^2^ Smoking pack years = number packs (25 cigarettes)/day × number years smoked; ^3^ Albumin:creatinine > 2.5 for men and > 3.5 for women.

**Table 2:** Peripheral and central blood pressure (BP), augmentation index (AIx) and pulse wave velocity (cfPWV) by treatment group over the 12 months study period.

	Intervention (*n* = 45)					Control (*n* = 47)					*p* Value		*p* Value after Adjustment for Baseline Value	
	Baseline	3 Months	6 Months	9 Months	12 Months	Baseline	3 Months	6 Months	9 Months	12 Months	Time Effect ^1^	Time × Treatment Effect ^1^	Time Effect ^1^	Time × Treatment Effect ^1^
Peripheral systolic blood pressure (mmHg)	127 ± 12	125 ± 14	126 ± 13	124 ± 11	127 ± 13	130 ± 15	131 ± 14	128 ± 14	126 ± 14	130 ± 15	0.053	0.56	0.72	0.46
Peripheral diastolic blood pressure (mmHg)	73 ± 11	71 ± 10	71 ± 10	72 ± 11	70 ± 10	72 ± 9	72 ± 11	71 ± 8	70 ± 10	73 ± 12	0.027	0.053	0.37	0.018
Peripheral pulse pressure (mmHg)	53 ± 12	53 ± 12	55 ± 14	52 ± 14	56 ± 15	58 ± 15	59 ± 14	57 ± 14	55 ± 13	57 ± 14	0.04	0.19	0.70	0.13
Central systolic blood pressure (mmHg)	126 ± 15	120 ± 14	119 ± 14	117 ± 10	119 ± 14	127 ± 16	124 ± 15	120 ± 11	120 ± 14	123 ± 13	0.001	0.74	0.62	0.19
Central diastolic blood pressure (mmHg)	83 ± 10	78 ± 10	79 ± 10	79 ± 10	79 ± 10	83 ± 10	81 ± 10	80 ± 9	79 ± 10	82 ± 11	0.001	0.63	0.98	0.51
Central mean arterial pressure (mmHg)	100 ± 11	94 ± 11	94 ± 11	94 ± 9	95 ± 10	101 ± 12	97 ± 10	96 ± 9	95 ± 11	98 ± 11	0.001	0.80	0.91	0.60
Central pulse pressure (mmHg)	42 ± 12	41 ± 11	40 ± 11	38 ± 10	40 ± 11	44 ± 14	43 ± 15	41 ± 10	41 ± 11	42 ± 11	0.003	0.71	0.23	0.03
Heart rate (bpm)	70 ± 11	68 ± 10	70 ± 11	72 ± 11	69 ± 11	71 ± 13	70 ± 12	70 ± 12	71 ± 13	70 ± 12	0.23	0.71	0.20	0.30
Central augmented pressure (mmHg)	9 ± 5	10 ± 7	11 ± 10	9 ± 7	10 ± 8	9 ± 4	9 ± 6	10 ± 6	10 ± 5	10 ± 6	0.02	0.19	0.007	0.17
Augmentation index (%)	20 ± 8	24 ± 15	25 ± 20	21 ± 15	22 ± 15	20 ± 7	20 ± 8	24 ± 12	23 ± 10	23 ± 11	0.002	0.23	0.02	0.55
Augmentation index at HR 75 (%)	18 ± 7	22 ± 14	23 ± 19	20 ± 14	20 ± 14	18 ± 7	19 ± 8	22 ± 11	22 ± 10	21 ± 11	0.007	0.36	0.03	0.67
cfPWV (m/s) ^2^	9.3 ± 1.9	9.2 ± 1.9	9.6 ± 2.0	9.5 ± 2.0	9.6 ± 2.1	9.7 ± 1.8	9.7 ± 1.8	9.7 ± 1.7	10.0 ± 1.9	9.9 ± 1.9	0.048	0.66	0.94	0.59
Pulse transit time (m/s)	61 ± 10	62 ± 8	61 ± 10	61 ± 9	60 ± 9	59 ± 10	58 ± 9	59 ± 9	57 ± 9	57 ± 9	0.045	0.86	0.74	0.29

^1^ Mixed effect modelling incorporating data from all time-points; ^2^ *n* = 78 (intervention *n* = 36; control *n* = 42).

**Table 3:** Change in weight and biochemistry by treatment group over the 12 months intervention period.

	Intervention Group (*n* = 45)					Control Group (*n* = 47)					*p* Value		*p* Value after Adjustment for Baseline Value	
	Baseline	3 Months	6 Months	9 Months	12 Months	Baseline	3 Months	6 Months	9 Months	12 Months	Time Effect ^1^	Time × Treatment Effect ^1^	Time Effect ^1^	Time × Treatment Effect ^1^
Weight (kg)	98.6 ± 17.9	97.9 ± 18.6	97.8 ± 16.9	98.4 ± 16.8	98.8 ± 17.7	97.2 ± 24.4	97.6 ± 24.5	96.5 ± 24.3	96.5 ± 24.7	96.9 ± 24.4	0.80	0.92	0.86	0.41
Total cholesterol (mmol/L)	4.0 ± 1.2	3.9 ± 1.0	4.0 ± 1.0	4.1 ± 0.9	4.0 ± 1.0	3.6 ± 1.0	3.7 ± 0.9	3.9 ± 1.1	3.8 ± 1.0	3.7 ± 0.9	0.42	0.60	0.32	0.65
HDL cholesterol (mmol/L)	1.2 ± 0.3	1.2 ± 0.3	1.3 ± 0.4	1.3 ± 0.3	1.2 ± 0.4	1.3 ± 0.4	1.3 ± 0.4	1.3 ± 0.4	1.3 ± 0.4	1.3 ± 0.4	0.19	0.89	0.85	0.20
LDL cholesterol (mmol/L)	2.2 ± 1.1	2.1 ± 0.9	2.2 ± 0.9	2.2 ± 0.8	2.2 ± 0.9	1.8 ± 0.7	1.9 ± 0.5	1.9 ± 0.8	1.8 ± 0.7	1.9 ± 0.8	0.99	0.57	0.11	0.41
Triglycerides (mmol/L)	1.1 ± 0.6	1.2 ± 0.6	1.2 ± 0.5	1.3 ± 0.6	1.2 ± 0.6	1.2 ± 1.2	1.2 ± 1.1	1.4 ± 1.6	1.3 ± 1.2	1.2 ± 1.1	0.43	0.95	0.62	0.59
Glucose (mmol/L)	7.6 ± 3.1	7.3 ± 2.8	7.2 ± 2.9	7.5 ± 3.1	7.4 ± 3.0	7.6 ± 2.9	7.3 ± 2.5	7.6 ± 3.3	7.2 ± 3.0	7.3 ± 2.2	0.90	0.71	0.31	0.07
hsCRP (mg/L) ^2^	1.7 ± 1.6	2.2 ± 2.6	2.0 ± 1.8	1.5 ± 1.1	1.7 ± 1.5	2.8 ± 2.7	2.6 ± 2.6	3.0 ± 2.9	2.3 ± 2.4	2.5 ± 2.6	0.016	0.91	0.83	0.97
HbA1c														
(%)	7.0 ± 1.2				7.3 ± 1.3	7.3 ± 1.6				7.2 ± 1.0	0.44	0.12	-	-
(mmol/mol)	53 ± 13				56 ± 15	56 ± 17				55 ± 11	0.44	0.12	-	-
MMP-7 (ng/mL) ^3^	3.3 ± 1.3				3.2 ± 1.2	3.3 ± 1.4				3.3 ± 1.4	0.92	0.58	-	-

^1^ Mixed effect modelling incorporating data from all time-points; ^2^ *n* = 64 (intervention *n* = 30; control *n* = 34); ^3^ *n* = 54 (intervention *n* = 27; control *n* = 27).

## Supplementary Materials

The following are available online at http://www.mdpi.com/2072-6643/8/6/382/s1, Table S1. Nutrient intake from the FFQ by treatment group, Table S2. Intake of food groups (g/day) from the FFQ by treatment group, Table S3. Urinary excretion data by treatment group.
