# Supplementary material for: Effect of Improving Dietary Quality on Arterial Stiffness in Subjects with Type 1 and Type 2 Diabetes: A 12 Months Randomised Controlled Trial
Source: Nutrients. 2016 Jun 21;8(6):382. doi: 10.3390/nu8060382 (PMC4924222; doi:10.3390/nu8060382)
Supplement: Supplementary file 1 [file nutrients-08-00382-s001.docx]

Supplementary Materials: Effect of Improving Dietary Quality on Arterial Stiffness in Subjects with Type 1 and Type 2 Diabetes: A 12 Months Randomised Controlled Trial

Kristina S. Petersen, Peter M. Clifton, Natalie Lister and Jennifer B. Keogh

**Table S1****.** Nutrient intake from the FFQ by treatment group.

| **Nutrient Intake** | **Intervention Group (*n* = 45)** | | | **Control Group (*n* = 47)** | | | ***p* Value** | |
| --- | --- | --- | --- | --- | --- | --- | --- | --- |
|  | **Baseline** | **3 Months** | **12 Months** | **Baseline** | **3 Months** | **12 Months** | **Time Effect ^1^** | **Time × Treatment Effect ^1^** |
| Energy (kJ/day) | 7995 ± 3086 | 7836 ± 3049 | 8188 ± 3162 | 7956 ± 3591 | 6999 ± 3231 | 7449 ± 3133 | 0.24 | 0.14 |
| Protein (g/day) | 94 ± 37 | 98 ± 37 | 103 ± 49 | 97 ± 42 | 92 ± 63 | 96 ± 46 | 0.69 | 0.35 |
| % E protein | 20 ± 3 | 21 ± 5 | 21 ± 4 | 21 ± 3 | 22 ± 4 | 22 ± 4 | 0.007 | 0.76 |
| Total fat (g/day) | 76 ± 35 | 72 ± 32 | 78 ± 35 | 77 ± 39 | 69 ± 42 | 71 ± 34 | 0.28 | 0.46 |
| % E total fat | 36 ± 6 | 35 ± 6 | 35 ± 6 | 36 ± 5 | 36 ± 6 | 36 ± 6 | 0.42 | 0.30 |
| Saturated fat (g/day) | 30 ± 14 | 28 ± 12 | 31 ± 15 | 29 ± 17 | 26 ± 18 | 27 ± 14 | 0.13 | 0.52 |
| % E saturated fat | 14 ± 3 | 13 ± 3 | 14 ± 3 | 13 ± 3 | 13 ± 3 | 14 ± 3 | 0.36 | 0.52 |
| Monounsaturated fat (g/day) | 28 ± 14 | 27 ± 13 | 28 ± 14 | 28 ± 14 | 26 ± 16 | 26 ± 13 | 0.55 | 0.58 |
| % E monounsaturated fat | 13 ± 3 | 13 ± 2 | 13 ± 3 | 13 ± 2 | 13 ± 3 | 13 ± 2 | 0.94 | 0.35 |
| Polyunsaturated fat (g/day) | 12 ± 6 | 11 ± 6 | 12 ± 6 | 13 ± 6 | 11 ± 6 | 12 ± 6 | 0.29 | 0.40 |
| % E polyunsaturated fat | 6 ± 2 | 5 ± 2 | 5 ± 2 | 6 ± 2 | 6 ± 2 | 6 ± 2 | 0.15 | 0.80 |
| Carbohydrate (g/day) | 188 ± 80 | 189 ± 76 | 191 ± 67 | 190 ± 79 | 154 ± 55 | 176 ± 76 | 0.09 | 0.005 |
| % E carbohydrate | 39 ± 7 | 41 ± 7 | 40 ± 6 | 41 ± 6 | 39 ± 7 | 40 ± 7 | 0.95 | 0.04 |
| Sugar (g/day) | 83 ± 37 | 92 ± 33 | 91 ± 32 | 77 ± 28 | 66 ± 25 | 74 ± 29 | 0.73 | 0.001 |
| Fibre (g/day) | 21 ± 8 | 23 ± 9 | 23 ± 8 | 22 ± 8 | 20 ± 7 | 22 ± 12 | 0.56 | 0.004 |
| Sodium (mg/day) | 2516 ± 1055 | 2509 ± 1230 | 2583 ± 1104 | 2622 ± 1352 | 2245 ± 1033 | 2410 ± 1125 | 0.22 | 0.09 |
| Potassium (mg/day) | 2909 ± 855 | 3251 ± 1034 | 3280 ± 1156 | 2964 ± 1052 | 2682 ± 988 | 2893 ± 1067 | 0.33 | 0.001 |
| Calcium (mg/day) | 946 ± 322 | 1052 ± 310 | 1084 ± 432 | 975 ± 386 | 864 ± 335 | 949 ± 353 | 0.25 | 0.001 |
| Magnesium (mg/day) | 305 ± 104 | 323 ± 114 | 331 ± 118 | 317 ± 111 | 283 ± 101 | 319 ± 146 | 0.35 | 0.007 |
| Alcohol (g/day) | 14 ± 23 | 11 ± 21 | 12 ± 21 | 10 ± 16 | 10 ± 16 | 9 ± 15 | 0.16 | 0.06 |

Values are mean ± SD; ^1^ Mixed effect modelling incorporating data from all time-points; % E percentage of total energy.

**Table S2.** Intake of food groups (g/day) from the FFQ by treatment group.

| **Dietary Intake (g/Day)** | **Intervention Group (*n* = 45)** | | | **Control Group (*n* = 47)** | | | ***p* Value** | |
| --- | --- | --- | --- | --- | --- | --- | --- | --- |
|  | **Baseline** | **3 Months** | **12 Months** | **Baseline** | **3 Months** | **12 Months** | **Time Effect** ^1^ | **Time × Treatment Effect** ^1^ |
| **Total breads and cereals** | 211 ± 108 | 207 ± 121 | 200 ± 106 | 233 ± 116 | 189 ± 79 | 221 ± 144 | 0.09 | 0.09 |
| **Total vegetables/legumes** | 157 ± 65 | 187 ± 92 | 180 ± 90 | 157 ± 64 | 151 ± 74 | 180 ± 77 | 0.038 | 0.056 |
| **Total fruit** | 283 ± 167 | 371 ± 194 | 284 ± 158 | 232 ± 133 | 202 ± 114 | 288 ± 150 | 0.09 | 0.001 |
| Fresh fruit | 220 ± 137 | 267 ± 136 | 232 ± 130 | 167 ± 105 | 161 ± 105 | 251 ± 151 | 0.03 | 0.001 |
| Canned fruit | 24 ± 57 | 50 ± 67 | 23 ± 42 | 12 ± 21 | 9 ± 21 | 14 ± 24 | 0.19 | 0.04 |
| Juice | 39 ± 58 | 54 ± 78 | 29 ± 48 | 53 ± 83 | 31 ± 50 | 23 ± 39 | 0.046 | 0.02 |
| **Total dairy** | 377 ± 184 | 451 ± 162 | 374 ± 163 | 393 ± 222 | 361 ± 197 | 463 ± 198 | 0.42 | 0.001 |
| Milk | 312 ± 180 | 344 ± 163 | 295 ± 140 | 321 ± 207 | 305 ± 182 | 392 ± 185 | 0.50 | 0.02 |
| Yogurt | 51 ± 51 | 95 ± 86 | 67 ± 63 | 60 ± 61 | 47 ± 56 | 57 ± 61 | 0.017 | 0.001 |
| Cheese | 14 ± 13 | 13 ± 10 | 12 ± 10 | 13 ± 12 | 9 ± 8 | 14 ± 12 | 0.08 | 0.09 |
| Total reduced fat dairy | 250 ± 219 | 277 ± 209 | 208 ± 182 | 251 ± 191 | 245 ± 194 | 287 ± 223 | 0.98 | 0.20 |
| Total full fat dairy | 76 ± 124 | 80 ± 146 | 99 ± 144 | 83 ± 204 | 69 ± 166 | 119 ± 220 | 0.22 | 0.66 |
| **Total meats and alternatives** | 209 ± 118 | 224 ± 123 | 223 ± 151 | 203 ± 106 | 220 ± 240 | 224 ± 170 | 0.38 | 0.98 |
| **Total extra foods** | 341 ± 454 | 270 ± 443 | 188 ± 223 | 254 ± 265 | 225 ± 271 | 223 ± 357 | 0.01 | 0.26 |
| Takeaway foods | 43 ± 36 | 38 ± 32 | 42 ± 41 | 54 ± 67 | 40 ± 52 | 33 ± 31 | 0.07 | 0.25 |
| Sweets | 49 ± 41 | 32 ± 29 | 37 ± 38 | 49 ± 51 | 30 ± 24 | 28 ± 28 | 0.001 | 0.71 |
| Savoury snacks | 14 ± 15 | 13 ± 11 | 12 ± 11 | 12 ± 13 | 9 ± 12 | 12 ± 14 | 0.29 | 0.53 |
| Alcoholic beverages | 235 ± 452 | 187 ± 438 | 97 ± 209 | 139 ± 220 | 146 ± 261 | 150 ± 354 | 0.22 | 0.04 |

Values are mean ± SD; ^1^ Mixed effect modelling incorporating data from all time-points.

**Table S3.** Urinary excretion data by treatment group.

| **Urinary Excretion** | **Intervention Group  (*n* = 45)** | **Control Group  (*n* = 47)** | ***p* Value** | |
| --- | --- | --- | --- | --- |
|  |  |  | **Time Effect** ^1^ | **Time × Treatment Effect** ^1^ |
| Spot sodium (mmol/L) | | | 0.005 | 0.35 |
| *Baseline* | 73 ± 39 | 78 ± 36 |  |  |
| *3 months* | 80 ± 39 | 84 ± 39 |  |  |
| *6 months* | 83 ± 43 | 74 ± 31 |  |  |
| *9 months* | 72 ± 39 | 84 ± 45 |  |  |
| *12 months* | 86 ± 43 | 96 ± 47 |  |  |
| Spot potassium (mmol/L) | | | 0.03 | 0.57 |
| *Baseline* | 70 ± 33 | 76 ± 38 |  |  |
| *3 months* | 80 ± 41 | 70 ± 39 |  |  |
| *6 months* | 76 ± 44 | 72 ± 39 |  |  |
| *9 months* | 65 ± 41 | 64 ± 35 |  |  |
| *12 months* | 68 ± 35 | 66 ± 35 |  |  |
| Spot creatinine (mmol/L) | | | 0.11 | 0.29 |
| *Baseline* | 9.4 ± 5.8 | 11.7 ± 7.1 |  |  |
| *3 months* | 10.7 ± 6.3 | 11.2 ± 7.5 |  |  |
| *6 months* | 11.4 ± 7.4 | 10.6 ± 5.6 |  |  |
| *9 months* | 8.8 ± 5.4 | 10.6 ± 7.5 |  |  |
| *12 months* | 8.8 ± 5.1 | 10.4 ± 6.3 |  |  |
| Sodium to potassium ratio | | | 0.015 | 0.40 |
| *Baseline* | 1.2 ± 0.8 | 1.3 ± 1.0 |  |  |
| *3 months* | 1.2 ± 0.6 | 1.5 ± 1.1 |  |  |
| *6 months* | 1.3 ± 0.8 | 1.4 ± 1.0 |  |  |
| *9 months* | 1.4 ± 1.0 | 1.6 ± 1.0 |  |  |
| *12 months* | 1.4 ± 0.7 | 1.8 ± 1.3 |  |  |
| Potassium to creatinine ratio | | | 0.16 | 0.35 |
| *Baseline* | 9.4 ± 5.0 | 7.5 ± 3.3 |  |  |
| *3 months* | 9.0 ± 5.2 | 8.0 ± 4.4 |  |  |
| *6 months* | 8.1 ± 4.6 | 7.5 ± 3.4 |  |  |
| *9 months* | 8.3 ± 4.3 | 7.0 ± 2.5 |  |  |
| *12 months* | 9.8 ± 8.6 | 7.3 ± 3.6 |  |  |
| Sodium to creatinine ratio | | | 0.11 | 0.24 |
| *Baseline* | 10.4 ± 6.8 | 8.1 ± 4.6 |  |  |
| *3 months* | 9.5 ± 5.4 | 10.0 ± 6.6 |  |  |
| *6 months* | 10.6 ± 9.8 | 8.9 ± 5.5 |  |  |
| *9 months* | 10.1 ± 6.1 | 10.8 ± 7.3 |  |  |
| *12 months* | 12.9 ± 12.6 | 11.2 ± 6.4 |  |  |

Values are mean ± SD; ^1^ Mixed effect modelling incorporating data from all time-points.
